# Probiotics, prebiotics, and synbiotics in childhood diarrhea

**DOI:** 10.1590/1414-431X2024e13205

**Published:** 2024-04-19

**Authors:** E.M.S. Martins, L.C. Nascimento da Silva, M.S. Carmo

**Affiliations:** 1 Laboratório de Patogenicidade Microbiana, Universidade Ceuma, São Luís, MA, Brasil; 2 Centro Universitário Dom Bosco, São Luís, MA, Brasil

**Keywords:** Diarrhea, Child, Probiotic, Prebiotic, Synbiotics, Supplement

## Abstract

Acute diarrhea is the second leading cause of morbidity and mortality attributed to infections in children under five years of age worldwide, with 1.7 million annual estimated cases and more than 500,000 deaths. Although hydroelectrolytic replacement is the gold standard in treating diarrhea, it does not interfere with the restoration of the intestinal microbiota. Several studies have searched for an adequate alternative in restructuring intestinal homeostasis, finding that treatments based on probiotics, prebiotics, and synbiotics are effective, which made such treatments increasingly present in clinical practice by reducing illness duration with minimal side effects. However, there are still controversies regarding some unwanted reactions in patients. The diversity of strains and the peculiarities of the pathogens that cause diarrhea require further studies to develop effective protocols for prevention and treatment. Here, we provide a descriptive review of childhood diarrhea, emphasizing treatment with probiotics, prebiotics, and synbiotics.

## Introduction

According to WHO estimates, diarrheal diseases are among the most common causes of malnutrition. They are the world's second leading cause of mortality in children under the age of 5 years, with around 1.7 million annual cases and more than 500,000 deaths. In Brazil, there are 4 million cases of acute diarrhea and more than 4,000 deaths annually according to the Mortality Information System (SIM) (1). In addition to a high risk of death, there is a risk of impaired physical and cognitive development in children (2).

Diarrhea is characterized by three or more episodes of softened or liquid stools per day. It may have different etiologies, such as infectious, dietary, allergic, functional, and inflammatory/autoimmune causes. The duration of bowel movements is classified as acute, persistent, secretory, or chronic (3-5).

Hydro-electrolytic replacement is the gold standard in the treatment of different diarrheal conditions, but it does not a have direct effect on microbiota restoration. Antibiotic therapy is not recommended in many infectious clinical pictures because the patient may continue to harbor the pathogen and evolve with intense dysbiosis (6).

In this context, probiotics, prebiotics, and synbiotics, which can be used as lyophilized drugs/suspensions or dietary supplements, emerge as adjuvant therapeutic and prevention approaches (7). Probiotics are live microorganisms that when consumed are beneficial to the host's intestinal health by rebuilding the intestinal microflora, modulating some intestinal characteristics, and optimizing resistance against pathogens. Prebiotics are non-digestible food components with the capacity of stimulating the growth and/or activity of specific microorganisms. To be considered a prebiotic, the component must be plant-derived, be composed of complex cells, be resistant to degradation by digestive enzymes, be fermented by bacterial colonies, and be osmotically active (8). Synbiotics are products composed of probiotics and prebiotics and whose synergistic actions enhance the beneficial effects of both components to promote the health of the host (9).

There is scientific evidence of the beneficial action of probiotics, prebiotics, and synbiotics in re-establishing intestinal homeostasis, restructuring the microbiota, and consequently improving the patient's clinical picture (10). However, some studies report adverse effects of increased bowel movements/constipation, bloating, flatulence, nausea, and even bacteremia with evolution to sepsis (11).

Because of the great diversity of microorganisms in the intestinal microbiota and their peculiar interaction with the host, which is influenced by factors such as age, lifestyle, nutrition, immunity, and previous chronic diseases, it is important to develop studies that can clarify the impact of the use of different probiotics on the drug-patient response and the attenuation/resolvability of diarrhea.

## Childhood diarrhea

Childhood diarrhea is a severe public health problem, and despite its several causes, the infectious form is still predominant. The high incidence rates, especially in socially disadvantaged regions, are intrinsically related to poor hygiene-sanitary conditions and basic sanitation (12).

The infection transmission is oral-fecal; the primary pathogens are viruses, bacteria, and intestinal parasites (13). Rotavirus, adenovirus, and norovirus are the most prevalent viral pathogens. In developing countries, enteric bacteria and parasites are most commonly found as an infectious cause in the summer. Among the bacterial pathogens are *Escherichia coli*, *Campylobacter* spp., *Shigella* spp., *Vibrio cholerae*, and *Salmonella enterica* (14).

On the other hand, diarrheal conditions of dietetic, allergic, functional, and inflammatory/autoimmune origin have been highlighted in recent years among the pediatric population due to advances in diagnostic techniques. In these cases, intrinsic host factors, including metabolism, genetics, and previous chronic diseases, are determinants (15).

**Figure 1:**
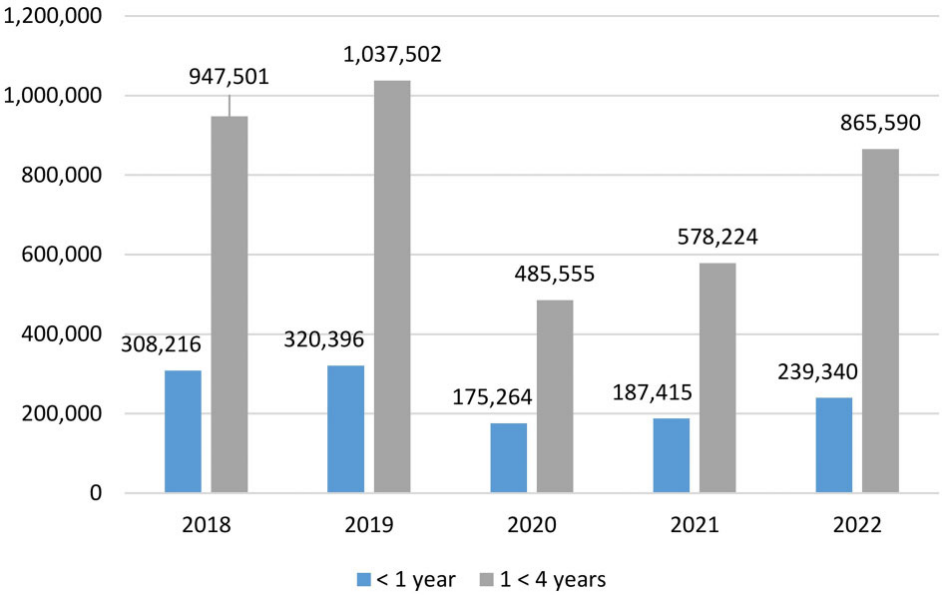
Cases of acute diarrheal diseases in Brazil according to the age group and year from 2018 to 2022.

In Brazil, the occurrence of acute diarrheal diseases must be reported to the Epidemiological Surveillance Information System for Acute Diarrheal Diseases (SIVEP DDA) through sentinel units implemented by the Brazilian Ministry of Health. The objective is the early detection of outbreaks, virulent and epidemic etiological agents, and situations of social vulnerability such as floods and droughts (16). According to DATASUS, from 2018 to 2022, 5,145,003 cases of acute diarrheal disease (ADD) were recorded among children under 5 years of age in Brazil (Figure 1). The mortality rate is around 4,000 deaths per year, with a higher infant mortality in patients under one year of age (17).

### Pathophysiology of diarrhea

According to the WHO, diarrhea is characterized by decreased stool consistency and increased frequency to three or more episodes in 24 h. Depending on the causative agent, the patient may present nausea, vomiting, abdominal pain, and fever (18).

The physiological absorptive capacity of the intestine fluctuates between 2-3 L/day. If inflammation/infection/injury or motility impairment occurs in the intestinal mucosa, water absorption capacity is decreased or secretion of water, electrolytes, and nutrients is increased, leading to diarrhea (19). Diarrhea can be caused by several factors including inflammatory diseases, fungal, bacterial, or viral infections, excessive consumption of sugar or other osmotic substances, allergic reactions to certain food, or even by absence of integrity of the absorptive surface of the intestinal mucosa (20).

According to Mahan et al. (21), diarrhea can be: i) Exudative: when there is damage to the intestinal mucosa that results in extravasation of mucus, fluid, blood, and plasma proteins, with a resulting accumulation of electrolytes and water; the release of prostaglandins and cytosines may also occur. Exudative diarrhea is provoked by conditions including Crohn's disease, ulcerative colitis, and infectious colitis; ii) Osmotic: when osmotically active solutes are present in the intestine but are poorly absorbed, such as with the excessive consumption of sugar; iii) Secretory: results from a bacterial or viral infection when excessive secretion of electrolytes and water by the intestinal epithelium occurs; iv) Malabsorptive absorption: resulting from pathological processes that cause impaired digestion or absorption, resulting in the excretion of nutrients and fat. Inflammatory bowel disease is an example; v) Drug-induced: induced by drugs that may cause an increase in bowel movements or intestinal motility.

The World Gastroenterology Organization Global Guideline (22) classifies diarrheal episodes as: i) Acute diarrhea, characterized by more than two liquid bowel movements in 24 h, lasting less than two weeks; ii) Persistent diarrhea: when the acute condition persists for more than 14 days and less than 4 weeks; iii) Chronic diarrhea: when diarrhea persists for more than 4 weeks; iv) Dysentery: when the presence of blood is evident to the naked eye.

Dehydration is the warning symptom of diarrhea and the first parameter to be monitored for possible correction of hydroelectrolyte markers, as this can lead to severe complications for the patient, including malnutrition, growth retardation, and delay in the child's cognitive development (23).

### Diagnosis of diarrhea

The diagnosis should be made based on the anamnesis, and physical and laboratory examinations. The main signs are decrease in fecal consistency, increase in bowel movement frequency, and at least 3 bowel movements in the previous 24 h. Some of the accompanying symptoms are abdominal cramps, nausea, vomiting, fever, and in some cases, hematochezia (24). The symptoms and their intensity vary depending on the etiological agent, as shown in Table 1. The pathogen may be identified by the history and clinical aspect of the patient, by visual/laboratory inspection of the feces, and by the incubation period of the pathogen, as described in Table 2.

**Table 1:** Main clinical characteristics of infectious diarrhea.

Pathogens	Clinical features					
	Abdominal pain	Fever	Evidence of inflammation in the stool	Vomit/Nausea	Heme-positive stools	Bloody stools
*Shigella*	++	++	++	++	+/-	+
*Salmonella*	++	++	++	+	+/-	+
*Campylobacter*	++	++	++	+	+/-	+
*Yersinia*	++	++	+	+	+	+
*Norovirus*	++	+/-	-	++	-	-
*Vibrio*	+/-	+/-	+/-	+/-	+/-	+/-
*Cyclospora*	+/-	+/-	-	+	-	-
*Cryptosporidium*	+/-	+/-	+	+	-	-
*Giardia*	++	-	-	+	-	-
*Entamoeba histolytica*	+	+	+/-	+/-	+/-	+/-
*Clostridium difficile*	+	+	++	-	+	+
*Shiga toxin-producing E.coli* (includes 0157:H7)	++	0	0	+	++	++

0: atypical/infrequent; +: occurs; ++: common; +/-: variable; -: not common. From the 2012 World Gastroenterology Organization Global Guideline (22; *J Clin Gastroenterol* 2013; 47: 12-20, doi: 10.1097/MCG.0b013e31826df662).

**Table 2:** Correlation between source of infection and main pathogens responsible for infectious diarrhea.

Source of infection	Etiologic agents of acute diarrhea			
Outbreak of foodborne infection	*Salmonella*	*Shiga toxin-producing E. coli* (STEC)	*Yersinia*	*Cyclospora*
Water transmission	*Vibrio*	*Giardia intestinalis*	*Cryptosporidium*	-
Seafood, crustaceans	*Vibrio*	Norovirus	*Salmonella*	-
Poultry	*Campylobacter*	*Salmonella*	*-*	-
Beef; raw seed sprouts	Shiga toxin-producing *E.coli* (STEC)	Enterohemorrhagic *E.coli* (EHEC)	*-*	-
Eggs	*Salmonella*	-	-	-
Mayonnaise and cream	*Staphylococcus*	*Clostridium perfringens*	*Salmonella*	-
Pies	*Salmonella*	*Campylobacter jejuni*	*Cryptosporidium*	*GIardia intestinalis*
Antibiotics, chemotherapy	*Clostridium difficile*	*-*	*-*	*-*
From person to person	*Shigella*	Rotavirus	*-*	*-*

From the 2012 World Gastroenterology Organization Global Guideline (22; *J Clin Gastroenterol* 2013; 47: 12-20, doi: 10.1097/MCG.0b013e31826df662).

The incubation period can vary from a few hours to days: i) <6 h: preformed toxin of *S. aureus* and *Bacillus cereus*; ii) 6-24 h: preformed toxin of *C. perfringens* and *B. cereus*; iii) 16-72 h: Norovirus, ECET, *Vibrio*, *Salmonella*, *Shigella*, *Campylobacter*, *Yersinia*, *Shiga toxin-producing E*. *coli*, *Giardia*, *Cyclospora*, and *Cyclosporidium*.

In cases of severe bloody, inflammatory, or persistent diarrhea, it is recommended that stool analysis and coproscopy be performed to promptly develop the management protocols to avoid the possible development of an outbreak or epidemic (25).

### Prevention

Because diarrheal diseases are related to inadequate sanitation, the most important action is to promote public policies not only to improve sanitation in cities with the distribution of drinking water within reach of all, but also educational programs that inform and guide the population about the importance of personal hygiene and food safety (26).

### Treatment

As dehydration is the first complication of acute diarrhea, the first recommendation is oral rehydration therapy for adequate replacement of hydroelectrolytic components and venous replacement, except in cases where the patient is already in a state of previous or installed dehydration or vomiting, or in children with ileus (27).

The Brazilian Ministry of Health warns of the importance of being aware of complications that may occur, such as vomiting, blood in the feces, reduced urination, and refusal to eat, in which case it is necessary to go to a health center (28).

Recent studies have suggested that probiotics can modulate the immune response by producing antimicrobial agents, competing for nutrients, and forming adhesion sites, an essential ally for the patient's recovery (29).

## Prebiotics

The first concept of prebiotics emerged in 1995 as a “non-digestible food ingredient that is beneficial to the host by selectively stimulating the growth or activity of one or a limited number of bacteria residing in the colon” (30). In 2015, the International Scientific Association for Probiotics and Prebiotics (ISAPP) updated the concept to “a substrate that is selectively used by host microorganisms conferring a health benefit”. Therefore, if a compound is harmful to the host, it cannot be considered prebiotic (31).

The definition of prebiotics could be confused with that of dietary fibers, if not for the characteristic of selectivity. Prebiotics must be selectively fermented by a colony of potentially beneficial bacteria (32). The most studied prebiotics are fructo-oligosaccharides (FOS), galacto-oligosaccharides (GOS), inulin, trans-galacto-oligosaccharides (TOS), oligofructo-inulin, lactulosa, oat fiber, germinated barley, hydrolyzed gum, resistant starch, *Plantago ovata*, beta-glucan, and pectin (33).

In addition, prebiotics should not be degraded by the upper portion of the digestive tract, and they must be highly fermentable, affecting the host microbiota and improving host health (34). Prebiotics such as inulin, FOS, GOS, and lactulose, when fermented, produce short-chain fatty acids (butyrate, propionate, and acetate) that reduce the decrease in colon pH by stimulating the growth of *Bifdobacteria* and *Lactobacilus*, in addition to acting as suppressors of pathogenic bacteria such as *Escherichia coli*, *Enterococcus faecalis*, and *Clostridium perfringers* (32).

## Probiotics

In 1908, the researcher and Nobel Prize winner Elie Metchnikoff linked the chronic consumption of dairy products to longevity, stating that the lactobacilli contained in these products act directly in reducing the toxins produced by intestinal bacteria and promoting host health (35).

Probiotics were first defined in 1965, being described as substances secreted by microorganisms that promote the growth of other microorganisms (36). Currently, the WHO still uses the definition formulated in 2001: probiotics are considered “living microorganisms that, when administered in adequate amounts, confer benefits to the health of the host” (37).

Although many studies have shown the beneficial effects of probiotics, so far, there is still a need for consensus on their medicinal use. In Europe, they are sold as nutritional supplements and the mention of beneficial influence on human health is prohibited. In countries such as Japan, Canada, and Switzerland, however, probiotics are widely prescribed to improve health (38).

Probiotics have several beneficial effects for maintaining and even restoring health in humans, such as anti-inflammatory effects (including improving the functionality of the intestinal barrier) and stimulating the production of antimicrobial peptides by host cells, thereby eliminating pathogenic bacteria (39).

**Figure 2:**
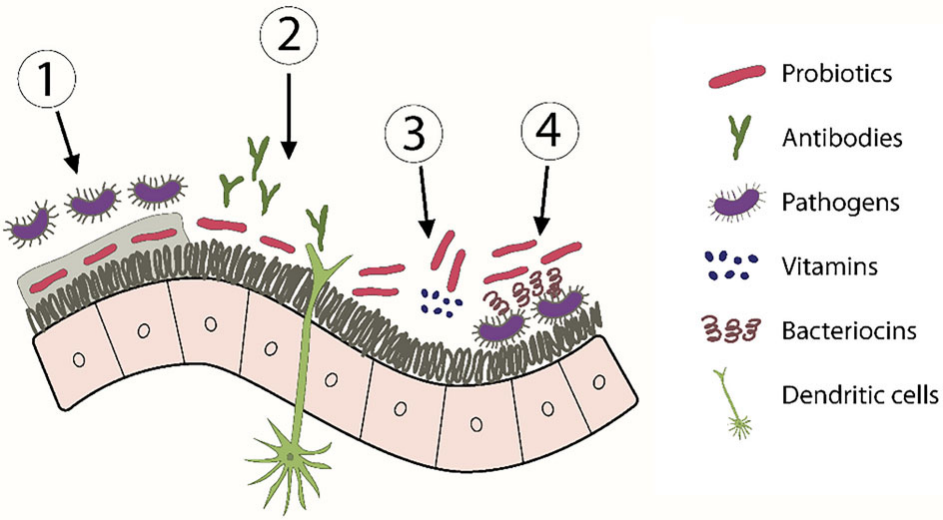
Mechanisms of action of probiotics. 1: Competitive exclusion; 2: Stimulation of the innate and adaptive immune response; 3: Increased nutrients bioavailability of nutrients; 4: Synthesis of bacteriocins and antimicrobials.

The mechanism of action of probiotics is intrinsically linked to their ability to adhere to host cells. Figure 2 shows the action of probiotics and some of their main functions, including the following: i) the ability to adhere to and proliferate on the intestinal mucosa; ii) stimulation of the growth of the commensal bacteria population and hindering (by exclusion); iii) the access of pathogenic bacteria to the intestinal lumen [1]. Probiotics possess immunomodulatory properties as they interact with intestinal epithelial cells, lymphocytes, dendritic cells, monocytes, and macrophages. They induce the release of immunoglobulin A by B cells, thereby enhancing mucosal immunity against the pathogens. The expression of interleukin (IL)-6, IL-10, and tumor necrosis factor (TNF)-β concomitant with the stimulation of immunoglobulin A is also observed [2].

Another important function is the ability to increase the quantity, bioavailability, and digestibility of nutrients [3]. An example is dairy probiotics that increase the amount of B vitamins. Through the release of enzymes, they promote the digestibility of proteins and fats and the release of free amino acids, lactic acid, and short-chain fatty acids with anti-inflammatory properties. Other probiotics produce butyric acid, which is capable of neutralizing dietary carcinogens.

Studies also suggest that some *Lactobacilli*, such as *Lactococcus lactis*, produce bacteriocins that act by preventing the growth and proliferation of pathogenic microorganisms, hindering the colonization of pathogens such as *E. coli* [4] (Figure 2) (40). The adhesion of probiotics to the cell wall promotes the strengthening of the intestinal mucosal barrier and they compete for the fermentation substrates, preventing pathogens from adhering (41).

Probiotics promote intestinal health by reducing the inflammatory process, creating an environment that is hostile to pathogens and conducive to the absorption of nutrients, thereby increasing their bioavailability (42).

There are a large number of microorganisms with probiotic potential. Among these, the most studied are the *Lactobacillus* and *Bifidobacterium* species, given that they have been found in all sections of the intestines of healthy people. Other species also considered probiotics are *Saccharomyces*, *Streptococcus*, and *Lactococcus* (43). The functionality of each probiotic, not only concerning the best microorganism for the treatment of diarrhea, but also the appropriate dose for each age group, is target of several studies (Table 3).

**Table 3:** Main probiotics used in the treatment of childhood diarrhea.

Probiotic or formula	Dose	Age group	Type of diarrhea	Findings
*B. lactis*	14.5×10^6^CFU/100 mL milk for 7 days	Less than 2 years	Essentially viral	Reduction of the duration of diarrhea (24 h) and the length of hospitalization (48 h)
*B. longum, B. lactis, L. acidophilus, L. rhaminosus, L. plantarum, and P. pentosaceus*	10^9^ CFU	3 months-7 years	Viral (Rotavirus)	Reduction of the duration of diarrhea (2 days) and vomiting (1 day)
*E. coli Nissle*	10^8^ CFU	1 month-4 years	Bacterial and viral	Effective in the treatment of diarrhea lasting more than 4 days
*L. rhamnosus* GG	10^9^ CFU	3 months-3 years	Bacterial and viral	Diarrhea and associated symptoms
	10^10^ CFU/250 mL of ORS^2^	1 month-3 years	Viral (Rotavirus)	Reduction in the duration of diarrhea
	10^9^ CFU	6 months-5 years	Viral (Rotavirus)	Reduction in the duration of diarrhea (18 h) and improvement of stool consistency.
*LGG, S. boulardii*, *B. claudii*; *L. delbrueckii var bulgaricus*, *S. thermophilus*, *L. acidophilus*, *B. bifidum*, and *E. faecium SF68.*	10^7^-10th CFU	3 months-3 years	Viral	Reduction in the duration of diarrhea by LGG (37 h) or mixing (45 h) and reduction in the number of bowel movements.
*L. paracasei ST 11*	5×10^9^ CFU/2 for 5 days	4 months-2 years	Bacterial and viral	Reduction of the frequency of bowel movements
*L.reuteri DSM 17938*	1×10^8^CFU + ORS for 5 days	3 months-5 years	Viral	Reduction in the duration of diarrhea (15 h)
	10^8^ CFU	3 months-5 years	Viral	Reduction in the duration of diarrhea (35 h) and length of hospitalization (24 h)
	4×10^8^	6 months-3 years	Viral	Reduction in the frequency and duration of diarrhea (1.2 days)
	10^8^ CFU/day	3 months	Bacterial	Reduction in enteropathogenic colonization
*L rhamnosus 19070-2* and *L.reuteri C+DSM 12246*	10^10^ CFU	9 months-4 years	Bacterial and viral	Reduction in the duration of diarrhea (40 h)
*S. boulardii*	250 mg/day	3 months-7 years	Bacterial, parasitic, and viral	Reduction in the duration of diarrhea (24 h) and length of hospitalization (24 h)
	500 mg/day for 5 days	3 months-5 years	Viral (Rotavirus)	Reduction in duration of diarrhea (29 h) and length of hospital stay (17 h)
	250 mg/1 or 2 days (depending on age)	3 months-2 years	Viral	Reduction in the duration of diarrhea (1.5 days) and frequency of bowel movements
*S. boulardii +* metronidazole	250 mg/2×daily for 7 days	10-12 years	Parasitic	Reduction in the duration of the time of elimination of blood in the stool

CFU: colony forming units. From do Carmo et al. 2018 (45; *Food Funct* 2018; 9: 5074-5095, doi: 10.1039/c8fo00376a).

A randomized clinical study with *L. rhamnosus* GG carried out with 1092 children demonstrates a significant reduction in diarrhea caused by rotavirus (44). Based on the analysis of Table 3, it is possible to confirm the effectiveness of some species (*L. rhamnosus* GG, *L. acidophilus*, *B. lactis*, *B. longum*, *L. plantarum*, *P. pentosaceus*, *L.reuteri*, *L. paracasei*, *S. boulardii*) in reducing both diarrhea duration and hospitalization length. In addition, *S. boulardi* plus metronidazole reduced the duration of blood elimination time in stools (45).

Currently, there are several products containing probiotics on the world market, some of which are easily found on the Brazilian market, such as Yakult (*Lactobacillus casei* Shirota), Activia (*Bifidobacterium lactis*), Enterogermina (*Bacilus clausii*), and Actimel (*Lactobacilli casei defensis*) (46).

## Synbiotics

Synbiotics combine prebiotics and probiotics, representing an alternative to increasing the amount and survival of probiotics in the digestive tract. Although some studies have shown the benefits of synbiotics, more evidence regarding species combination, microbial quantity, and other characteristics is needed to prove their effectiveness, because there must be a synergistic action between the substrate and the microorganism to result in a benefit for the host (10).

The main synbiotics studied are: i) *Lactobacillus plantarum* 299 + 10 g oat fiber; ii) *Lactobacillus sporogens* + fructo-oligosaccharides; iii) Synbiotic 2000: 10 CFU each of *Pediococcus pentoseceus* 5-33:3, *Leuconostoc mesenteroides* 32-77:1, *Lactobacillus paracasei sp*. *paracasei* 19, *Lactobacillus plantarum* 2362, and 2.5 g betaglucans, inulin, pectin, and resistant amide; iv) Synbiotic 2000 Forte: 10 CFU of *Pediococcus pentoseceus* 5-33:3, *Leuconostoc mesenteroides* 32-77:1, *Lactobacillus paracasei* sp. *paracasei* 19, *Lactobacillus plantarum* 2362, and *2.5* g of inulin, oat fiber, pectin, and resistant starch; v) Oligofructosa + inulin (SYN1) *+ Lactobacillus rhamnosus* GG and *Bifidobacterium lactis* Bb12; vi) Golden Bifid: *Bifidobacterium bifidum*, *Lactobacillus bulgaricus*, *and Streptococcus thermophilus* with FOS.

The studies indicate that the combination of probiotics and prebiotics (synbiotics) is more effective in reducing the duration of diarrhea and hospitalization compared to treatment using isolated strains. For instance, the treatment based on *Saccharomyces boulardii* and *Bifidobacterium* was more effective than the single administration of *Lactobacillus* (47).

The evaluation of the efficiency of probiotics or synbiotics is not straightforward because, given the expressive heterogeneity concerning the probiotic to be used, the need or not for the combination with prebiotics, the best combination to be used (synbiotic), the form of administration, the amount to be administered, and the correct dose of colony-forming units, in-depth study is necessary about all these nuances before administration to the patient so that no undesirable effects occur (48). Many studies involving the use of probiotics alone or in association with prebiotics have been and are being gradually developed. The existence of numerous strains and their interaction with the different existing pathogens and those that emerge daily makes it necessary to carry out further studies.

Although negative or null studies are more difficult to publish, these data are equally important and must be taken into consideration so that a detailed protocol can soon be developed on the types of bacteria with the quantification of colonies necessary for a beneficial influence, the consequences of bacterial interaction with prebiotics, and their effectiveness against certain pathogens and disease states, containing not only the benefits but the possible undesirable reactions that may occur.

## Final considerations

Although having several etiologies, infectious diarrhea is the most common diarrheal disease in children under five years of age and is a common condition in underdeveloped and developing countries, such as Brazil. In the pediatric routine, there are still many difficulties regarding the differential diagnosis between the types of diarrhea, which is associated with the emergency of improving the clinical picture, resulting in the inappropriate/indiscriminate use of antibiotics and the risk of a more critical evolution.

In this context, prebiotics, probiotics, and synbiotics as supplementary therapy have gained importance in the hospital routine since they help to recompose intestinal microbiota and reduce selective pressure on pathogenic microorganisms. On the other hand, some studies have reported unwanted effects, the extent and intensity of which vary according to various factors intrinsic to the host. This threshold has not yet been well elucidated in the literature.

Therefore, research evaluating the impact of the use of different prebiotics, probiotics, and synbiotics in the face of the most diverse diarrheal conditions is essential for a better understanding of the role that these supplements can play in patients, considering their physiological, nutritional, immunological, and clinical particularities.
